# A review of the human vs. porcine female genital tract and associated immune system in the perspective of using minipigs as a model of human genital *Chlamydia* infection

**DOI:** 10.1186/s13567-015-0241-9

**Published:** 2015-09-28

**Authors:** Emma Lorenzen, Frank Follmann, Gregers Jungersen, Jørgen S. Agerholm

**Affiliations:** Section for Veterinary Reproduction and Obstetrics, Department of Large Animal Sciences, Faculty of Health and Medical Sciences, University of Copenhagen, Copenhagen, Denmark; Chlamydia Vaccine Research, Department of Infectious Disease Immunology, Statens Serum Institut, Copenhagen, Denmark; Section for Immunology and Vaccinology, National Veterinary Institute, Technical University of Denmark, Copenhagen, Denmark

## Abstract

Sexually transmitted diseases constitute major health issues and their prevention and treatment continue to challenge the health care systems worldwide. Animal models are essential for a deeper understanding of the diseases and the development of safe and protective vaccines. Currently a good predictive non-rodent model is needed for the study of genital chlamydia in women. The pig has become an increasingly popular model for human diseases due to its close similarities to humans. The aim of this review is to compare the porcine and human female genital tract and associated immune system in the perspective of genital *Chlamydia* infection. The comparison of women and sows has shown that despite some gross anatomical differences, the structures and proportion of layers undergoing cyclic alterations are very similar. Reproductive hormonal cycles are closely related, only showing a slight difference in cycle length and source of luteolysing hormone. The epithelium and functional layers of the endometrium show similar cyclic changes. The immune system in pigs is very similar to that of humans, even though pigs have a higher percentage of CD4^+^/CD8^+^ double positive T cells. The genital immune system is also very similar in terms of the cyclic fluctuations in the mucosal antibody levels, but differs slightly regarding immune cell infiltration in the genital mucosa - predominantly due to the influx of neutrophils in the porcine endometrium during estrus. The vaginal flora in Göttingen Minipigs is not dominated by lactobacilli as in humans. The vaginal pH is around 7 in Göttingen Minipigs, compared to the more acidic vaginal pH around 3.5–5 in women. This review reveals important similarities between the human and porcine female reproductive tracts and proposes the pig as an advantageous supplementary model of human genital *Chlamydia* infection.

## Table of contents

1. Introduction

2. Methods

3. The female reproductive cycles

4. The female genital tract in pigs and humans

4.1 Gross anatomy

4.2 Microscopic anatomy

4.2.1 Vagina

4.2.2 Cervix

4.2.3 Uterus

4.2.4 Fallopian tubes

4.3 Anatomical and histological differences of relevance for a *Chlamydia* model

5. Genetics

6. The porcine immune system compared to the human immune system

6.1 The genital mucosal immune system

6.1.1 Distribution of immune cells in the genital tract tissue

6.1.2 The humoral genital immune response

6.2 Immunological differences of relevance for a *Chlamydia* model

7. The vaginal flora and pH

8. Important differences between rodents and minipigs

9. Conclusions

10. List of abbreviations

11. Competing interests

12. Authors’ contributions

13. Authors’ information

14. References

## 1. Introduction

Animal models are essential for gaining new insight into disease mechanisms of human genital diseases and the development of new prophylactic strategies and treatments [1]. Predominantly rodents are used as models, within pre-clinical research, with mice often being the animal of choice [2,3]. Rodent models have clear advantages both regarding practical issues, by being small and easy to handle, and economically affordable [2]. Furthermore, several genetically modified knockout strains are easily accessible, creating a unique opportunity to study the role of specific mediators in the immune response [4,5].

However, when evaluating animal models, different parameters are important to consider depending on the purpose of the model [6]:

- Face validity; how well is the biology and symptoms of the human disease mimicked by the model.
- Predictive validity; how well is the effect of a drug/compound or treatment mimicked by the model.
- Target validity; how similar a role the target of interest plays in the model compared to humans.

Despite the many advantages of rodent models, rodents show a number of differences to humans in terms of size, anatomy, physiology and immunology that do not always allow them to mimic the human course of infection and immune response [4,5,7,8]. The face validity and predictive validity is therefore prone to be insufficient, leaving a strong need for an intermediate and reliable model for the study of female genital tract (FGT) infections and the development of appropriate vaccines against them [9,10]. Non-human primates (NHP) are the animals most closely related to humans and therefore likely to show the greatest face- and predictive validity. However, due to ethical concerns and costly experiments associated with studies in NHP, there is a need for an intermediate pre-clinical/advanced non-rodent animal model.

The pig has become an increasingly popular model, especially within the fields of atherosclerosis and diabetes research, because of its physiological and anatomical similarities to humans [11-13]. Pigs of reduced body size such as the Göttingen Minipigs offer a great advantage by having a smaller size at sexual maturity and a lower growth rate than conventional pigs [14]. Furthermore, such breeds are available as specific pathogen free from specialized breeding companies [15]. Wherever possible, this review will focus on the minipig, since this has been the experimental animal of choice in our research. Despite the physical size, there are no studies reporting any physiological differences between minipigs and conventional pigs. Furthermore, Göttingen Minipigs are partly derived from German Landrace pigs [15].

It has recently been shown that pigs are susceptible to *Chlamydia trachomatis,* the agent causing human genital *Chlamydia*, and that pigs are suitable models for the study of *Chlamydia* pathogenesis and evaluation of vaccine candidates [16]. To evaluate the pig as a model of human genital *Chlamydia* and to be able to interpret and extrapolate results critically and reliably, it is important to understand the morphological and functional similarities and differences between the human and porcine female reproductive systems. The purpose of this review is to provide the basis for this understanding.

## 2. Methods

The PubMed database [17], Google Scholar [18] and CAB ABSTRACTS database were searched, with the following keywords: Pig/swine/porcine, genital tract/reproductive tract/vagina/cervix/uterus/uterine body/uterine horn/Fallopian tubes, immunology/immune response/immunity, mucosal immunity/immune response, estrous cycle/menstrual cycle/ sex hormone regulation immunity, pig model/porcine model/animal model, sexually transmitted disease/genital infections, vaginal microbiota/flora/ecosystem.

Due to the very limited numbers of original published papers within the search criteria no year limit was applied. The articles found were in the first line selected based on the abstract content, hereafter the selected articles were evaluated in detail and based on relevance for this review and on the quality of the study, articles were included in this review. Studies on pregnancy immunology/embryology were not included.

## 3. The female reproductive cycles

**Figure 1:**
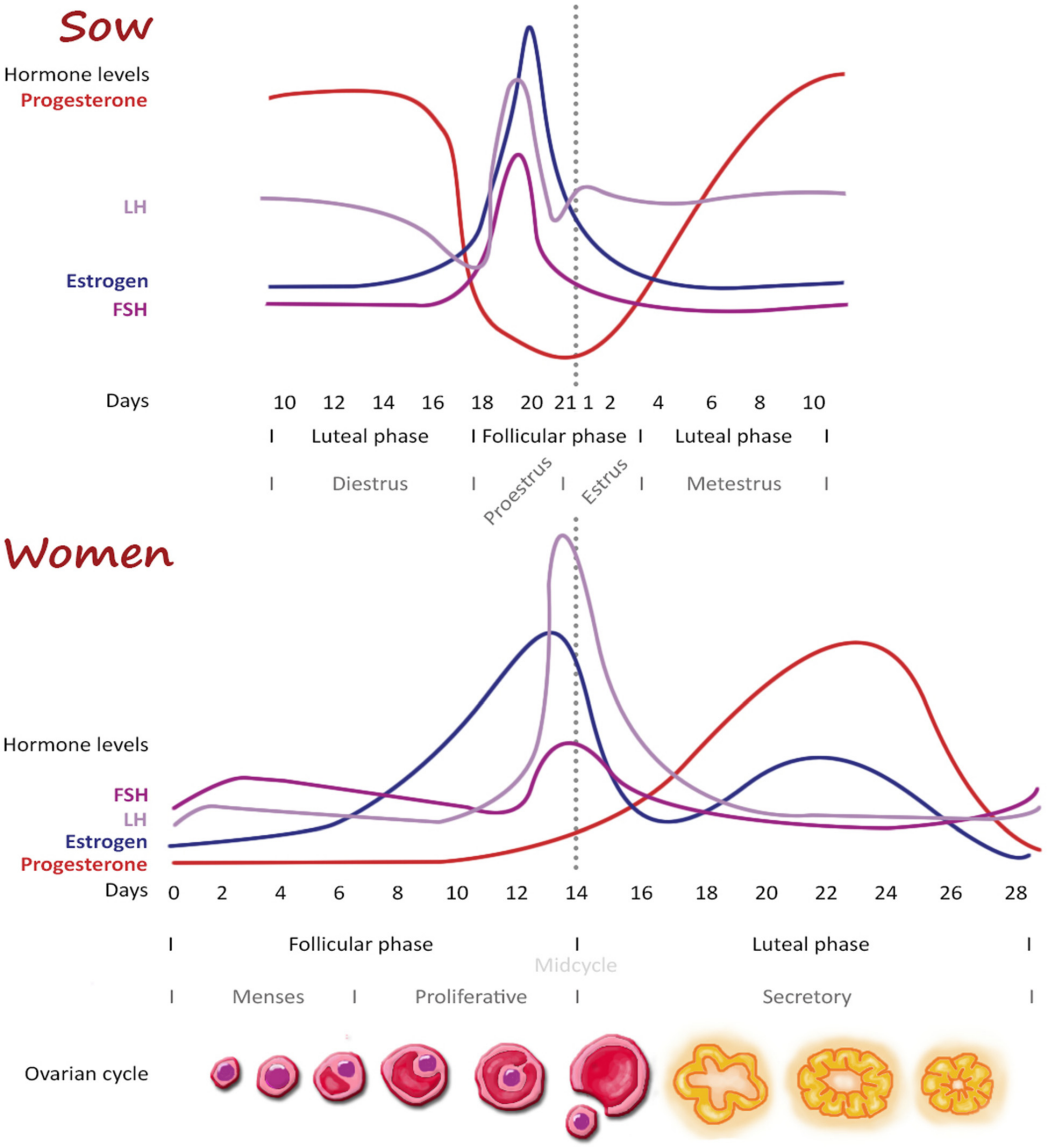
**Comparison of the hormonal reproductive cycles in women and pigs.** The estrous cycle in pigs begins and ends with ovulation/estrous [20,86]. The menstrual cycle in women begins and ends with the start of menses, with the ovulation in the middle of the cycle [19]. Otherwise, the length of the cycle and the hormonal fluctuations are very similar.

In women, the reproductive cycle (menstrual cycle) is described according to the gonadal activity or endometrial changes [19]. In pigs, the reproductive cycle (estrous cycle) is classified by the sexual behavior; estrus, where the pig is sexually receptive, or non-estrus [20]. Both of the cycles can be described with two phases; the luteal and the follicular phase, separated by ovulation (Figure 1).

In the pig, a significant follicle growth occurs during the luteal phase (i.e. the follicular phase overlaps the luteal phase), resulting in a slightly shorter cycle (19–21 days) than in women, where the two phases are more stringent separated and the cycle therefore lasts 28 days [19,20]. However, the mean length is very similar between pigs and women.

The menses/menstruation, a bloody uterine discharge, is specific for humans and some primates, usually lasts 3–7 days and is related to the beginning of the follicular phase [19]. Both women and pigs are spontaneous ovulators and continuously cycling [21]. A comparison of the changes in the reproductive hormones during the reproductive cycles is shown in Figure 1.

Both hormonal cycles are under control of the hypothalamic-pituitary-ovarian axis [19,20]. If no pregnancy occurs during an estrous cycle in the pig, the non-pregnant uterus secretes prostaglandin F2α (PGF-2α), which makes the corpus luteum regress (luteolysis) [20]. In women, the mechanism behind luteolysis is a bit more unclear, however, it is suggested that intraluteal PGF-2α plays a luteolysing role [22]. The important differences between the porcine estrous and human menstrual cycles are summarized in Table 1 together with the same parameters in primates and mice, to show the level of similarity compared to these species.

**Table 1:** **Comparison of reproductive-cycle parameters in women, non-human primates, minipigs and mice**

	**Women (menstrual)** [25,87]	**Non-humane primates (menstrual)** [88,89]	**Minipigs (estrous)** [11,20,90]	**Mice (estrous)** [84,91]
Cyclicity	Continuous cycling	*Baboons*: continuous cycling in captivity *Rhesus Macaque*: seasonal poly-oestral.	Continuous cycling	Continuous cycling
Age of sexual maturity	12.9 years	3 years	4–6 months	6–8 weeks
Length of cycle	28 days	28–33 days (highly variable)	19–21 days	3–5 days (highly variable)
Follicular/luteal phase	10–14 days/12–15 days	8 days/19 days	5–6 days/15–17 days	2 days/2–3 days
Luteolysis inducer	Ovarian PGF_2α_	Ovarian PGF_2α_	Uterine PGF_2α_	Uterine PGF_2α_
Endometrial sloughing/menstruation	Yes	Yes	No	No

## 4. The female genital tract in pigs and humans

### 4.1. Gross anatomy

**Figure 2:**
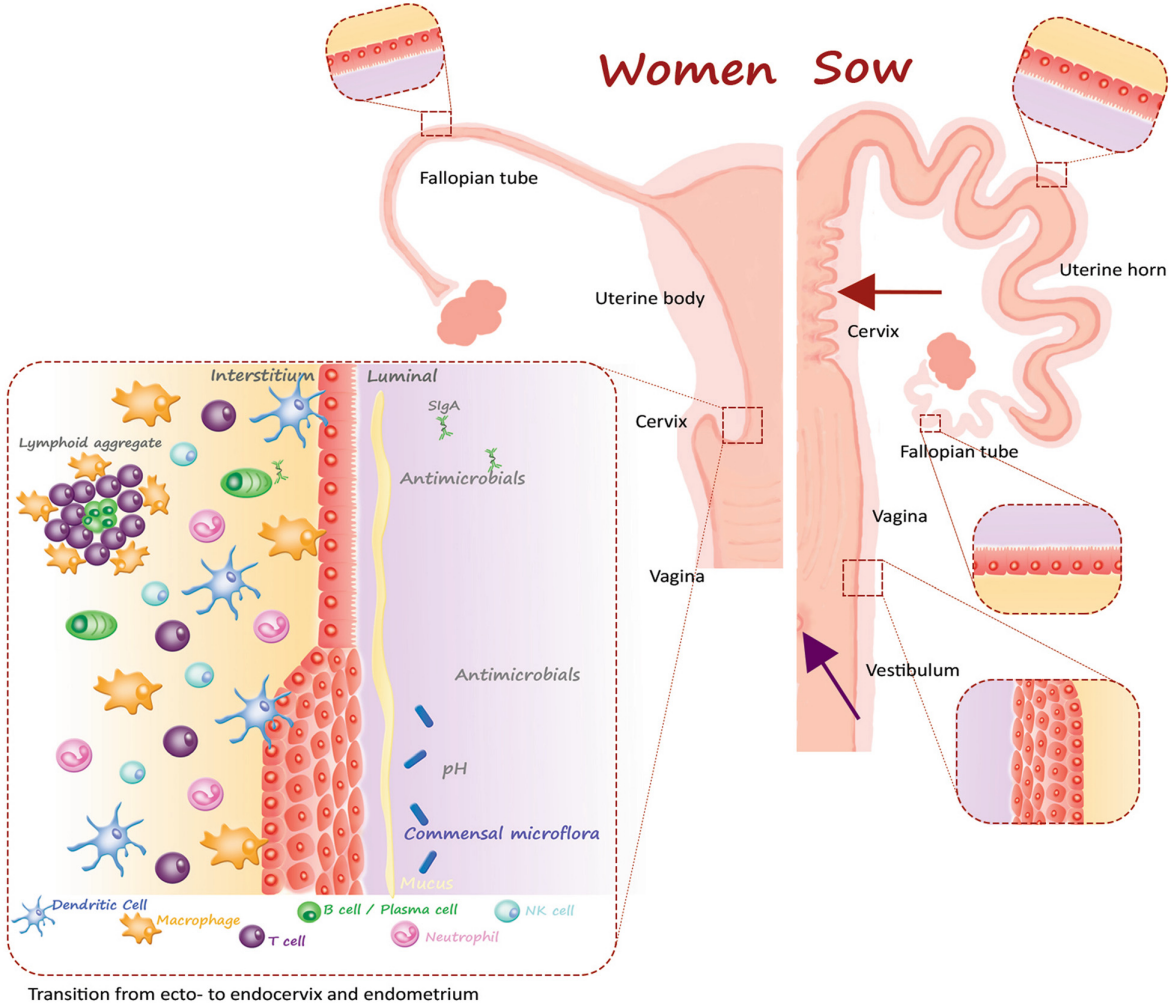
**Comparison of the gross anatomy and epithelium in the genital tract in women and pigs.** The porcine uterus differs macroscopically from the human simplex uterus by having bilateral horns (bicornuate) [23]. The porcine cervix displays a characteristic feature, not found in women; the cervical pulvini (red arrow) [23]. Furthermore, the porcine urethra opens on the ventral surface of the vagina (purple arrow) creating an urogenital sinus that opens to the outside through the common urogenital orifice [23]. In women, the urethra and vagina have its own separate openings to the outside [19]. Otherwise the porcine vagina is similar to the human one [92]. The human cervix is divided into the ectocervix that protrudes into the vaginal canal and the endocervix, creating the cervical lumen. An example of the local immune system in the female genital tract is shown at the transition between the ecto- and endocervix.

The porcine uterus differs from the human by being bicornuate [23] (Figure 2). The bicornuate elongation of the uterine body into two uterine horns creates a longer distance from the porcine cervix to the entrance of the Fallopian tubes than in women. In women the uterine body is approximately 7 cm long [24] while in a 1-year-old sexually mature Göttingen minipig gilt, each horn is an average 37.2 ± 5.9 cm long (mean ± SD, *n* = 12, unpublished data).

The cervix in Göttingen Minipigs is an average 7.5 ± 0.85 cm long, whereas the human cervix is around 2–3 cm [25]. The porcine cervix displays a characteristic feature, not found in women; the *pulvini cervicales* [23], which are a number of interdigitating prominent solid mucosal folds and protrusions throughout the length of the porcine cervix. Furthermore, the porcine urethra opens on the ventral surface of the vagina, creating a urogenital sinus that opens to the outside through the common urogenital orifice [11,14]. In women, the urethra and vagina have separate openings [19].

The vagina in women is approximately 7 cm along the anterior curvature and 9 cm along the posterior curvature [25]. In Göttingen Minipigs the vagina is an average 13.8 ± 0.9 cm (mean ± SD, *n* = 12, unpublished data).

The Fallopian tubes are 7–14 cm long and 0.5–1.2 cm in external diameter in women [26] and an average 17.3 ± 2.7 cm long and 0.4–0.5 cm in diameter (mean ± SD, *n* = 12, unpublished data) in 1-year-old Göttingen Minipigs.

### 4.2. Microscopic anatomy

Histology is a very important tool in the evaluation of pathological changes in animal models. Therefore, it is important to understand morphological differences between pigs and humans [12]. Generally, and common for both pigs and humans, the wall of the FGT consists of three layers: the mucosal, the muscular and the outer serosal layers [21]. The *tunica mucosa* facing the lumen (the endometrium), is built by the inner *lamina epithelialis*, *lamina propria* (connective tissue) and the *tela submucosa*. The muscular layer (*tunica muscularis*) is built by *stratum circulare* and *stratum longitudinale*. The outer *tunica serosa* (the perimetrium), facing the peritoneal and pelvic cavities, is built by a *lamina propria* and *lamina epithelialis* [21]. In the peritoneal cavity the *lamina epithelialis* of the *tunica serosa* has a simple squamous epithelium (visceral layer of the peritoneum) and in the pelvic cavity only loose connective tissue (adventitia) [21].

#### 4.2.1. Vagina

The vagina is the entry site for most sexually transmitted diseases and therefore of great importance when comparing the pig model with humans [12]. The vaginal *lamina epithelialis* is made by non-keratinized stratified squamous epithelium and forms longitudinal folds called *rugae* in both women and pigs [12,27]. The porcine vaginal epithelium undergoes cyclic alterations reaching a maximum thickness in the late proestrus [21]. The *lamina propria* consists of vascularized fairly dense connective tissue with no glands or mucosal muscular layer in both pigs and humans [21].

The vaginal mucosa is moisturized with secretions from the cervix. Cranially the porcine vagina is covered by a typical *tunica serosa* (i.e. loose connective tissue covered by the mesothelium) while caudally, a *tunica adventitia*, consisting of loose connective tissue is present. Both *tunica serosa* and *adventitia* contain large blood vessels, extensive venous and lymphatic plexuses and numerous nerve bundles and ganglia [21,28]. In women, the vagina is externally covered by adventitia, primarily built with elastic fibers attaching the vagina to the surrounding connective tissues and organs [27]. *Tunica muscularis* is also similar for pigs and humans with an inner layer of circularly arranged smooth muscle cells and an outer longitudinal layer, however the pig can have a thin layer within the circular layer with longitudinally arranged fibers [21,27]. Studies have furthermore shown that the porcine vaginal permeability barrier, which is based on the lipid composition and intercellular lipid lamellae in the epithelium, closely resembles that of humans [12].

#### 4.2.2. Cervix

The porcine cervix has a thick, muscular wall rich in elastic fibers [21,23], whereas the human only contains small amounts of smooth muscle and therefore mainly consists of dense connective tissue and elastic fibers [27].

The cervical *lamina epithelialis* differs between humans and pigs. In women the ectocervix has non-keratinized stratified squamous epithelium and the transformation zone separates it from the endocervix with a simple columnar epithelium [27]. In pigs, more than 90% of the cervix may have a vaginal type of epithelium with stratified squamous epithelium that undergoes cyclic alterations. The porcine cervical epithelium changes between simple columnar, pseudostratified and stratified squamous epithelium, with primarily columnar in diestrus and primarily stratified in estrus [21].

Common for both species is the simple columnar epithelium, which is mucinous with mucus secreting goblet cells. The amount of mucus secreted depends on the cycle stage with an increased amount during estrus in pigs and midcycle in women (around ovulation). Much of the mucus passes to the vagina. Similarly the epithelium increase in thickness and edema develops during proestrus and estrus [21]. After ovulation the secretion decreases and the mucus becomes thicker [21].

#### 4.2.3. Uterus

The human myometrium (*tunica muscularis*) is built by three muscular layers. The thick middle layer (*stratum vasculare)* contains many large vessels [27]. This highly vascularized and well-innervated *stratum vasculare* is, however, indistinct in the pig [21]. A *tela submucosa,* with dense irregular connective tissue, is not present in the uterus in women, where the epithelium with *lamina propria* lie closely applied to the myometrium [27].

The epithelium is simple columnar in both women and pigs, but in the pig it increases significantly in height during estrus and can turn into high pseudostratified columnar epithelium [21,29]. The endometrium and structure of the epithelial cells in women are also highly responsive to the hormonal changes and the thickness of the endometrium increases during the late proliferative phase [21,30].

The endometrium in pigs and women can be characterized by two zones or layers; the superficial functional layer (*stratum functionale*) and the deeper basal layer (*stratum basale*). The functional layer undergoes cyclic changes and degenerates partly or completely after pregnancy and estrus in the pig [21]. In humans, the degenerated tissue is shed during menstruation [27]. In contrast to women, the pigs’ basal layer is more cellular and fibrous. It remains during all cyclic stages and is the source for restoration of the functional layer [21,27].

The uterine epithelium in pigs and women contains both ciliated cells and non-ciliated secretory cells [21] and branched and coiled (endometrial) glands that extend into the *lamina propria* [28]. In women, these glands are short and straight in the proliferative (follicular) phase and long and coiled in the secretory (luteal) phase [30]. In the porcine endometrium, growth and branching of the glands are stimulated by estrogen and the coiling and copious secretion by progesterone [21,29].

#### 4.2.4. Fallopian tubes

The Fallopian tubes are of special interest in genital *Chlamydia* research, as they represent the site of infection, where sterilizing pathology develops in women [31]. The mucosa at the Fallopian tubes is folded into longitudinal folds (*plicae*) and the epithelium has non-ciliated secretory cells and ciliated cells that aid in moving the sperm upwards and the ovum downwards. The mucosal plicae in the ampulla have secondary and sometimes tertiary folds creating a complex system of epithelial-lined spaces. The epithelial lining is made of a single layer of columnar epithelial cells which sometimes is pseudostratified in pigs [21,32]. The epithelium undergoes cyclic changes with the greatest height and ciliation in the late follicular phase, and atrophy together with loss of cilia in the luteal phase [30].

The Fallopian tube in both pigs and humans can be separated into three parts; the *isthmus*, which is communicating with the uterus, the *ampulla* (the middle thin walled part), and the *infundibulum* that has fimbriae to catch the oocyte, when it is released into the peritoneal cavity during ovulation. The human Fallopian tubes furthermore have an extra compartment called the intramural part. Fertilization will take place in the *ampulla* in both pigs (caudal *ampulla*) and humans [21,27].

### 4.3. Anatomical and histological differences of relevance for a *Chlamydia* model

The slight anatomical differences in the pig are important to consider when choosing the inoculation route and when evaluating the ascending capacity of an infection. The porcine cervical *pulvini* make the access from the vagina to the uterus complicated in pigs and should be considered when choosing the inoculation method. Furthermore, the longer uterine body, in terms of uterine horns, is an important factor for the face validity of the pig model in evaluating ascending infections reaching the Fallopian tubes. In sexually immature conventional pigs inoculation with *C. trachomatis* SvE resulted in an ascending infection with bacterial replication in the Fallopian tubes [16].

A clear benefit of the porcine anatomy is the human-like prominent Fallopian tubes in the pig that potentially allows studying the tubal pathology induced by a *C. trachomatis* infection.

Since the columnar epithelial cells are the target cells for the *C. trachomatis* [16,33] it is important to be aware of the slightly different localization of the target cells. In women the columnar epithelial cells are found together with the transitional cells found in the endocervix and upper FGT [34]. In the pig, the cervix is dominated by stratified squamous epithelium and columnar cells are only consistently found in the porcine uterus [21,35], and therefore not at the vagino-cervical transition as in women. It is therefore recommended to inoculate pigs directly into the uterus.

## 5. Genetics

The majority of genes expressed in porcine female reproductive tissues are expressed in human FGT as well [36]. As further eluted to below, pigs share significantly more immune-system related genes and proteins with humans than mice do [37].

## 6. The porcine immune system compared to the human immune system

The porcine immune system is well characterized and highly resembles that of humans [11,36], although there are some differences. One of the differences is the anatomy of the lymph nodes, which are inverted in pigs [38]. The inverted lymph node structure only affects the lymphocyte migration through the lymph node. Porcine lymphocytes mainly leave the lymph node through high endothelial venules instead of efferent lymph vessels, as they do in humans [21,38,39]. Otherwise the physiology and immunologic reactions of the B and T cell areas in the lymph nodes do not differ [21,38].

Most of the protein mediators of the immune system are present with the same structure and function in humans and pigs and most of the immune cells identified in both species are similar [36,40]. The distribution of leukocytes in the blood is very similar in pigs and humans with a high percentage of neutrophils [41], however, within the lymphocyte populations, pigs have a higher proportion of CD4^+^CD8^+^ double positive T cells and ƴδ T cells in the blood. Otherwise the distribution of the different lymphocyte populations in pigs and humans is quite similar [11,36,40,42] as summarized in Table 2.

**Table 2:** **Lymphocyte subsets and antibodies in serum in humans and pigs**

	**Lymphocytes** [42,93,94]						**Antibodies (in serum)** [11,37,70]				
	**B cells**	**T cells (ƴd)**	**T cells (αβ)**			**NK cells**	**IgM**	**IgG**	**IgA**	**IgE**	**IgD**
Humans	18–47%	2–8%	28–59% (CD4+)	13–32% (CD8+)	<3% (CD4 + CD8+)	2–13%	5–10%	80%	10–15%	<0,05%	0,2%
Pigs	8–18%	9–19%	25–27% (CD4+)	27–32% (CD8+)	10–13% (CD4 + CD8+)		10–12%	80–85%	5–12%	<0,01%	Not described

The major histocompatibility complex (MHC) system in pigs, called the swine leukocyte antigen (SLA) system is very similar to the human leukocyte antigen system, in terms of polymorphic loci, haplotypes and differentiated expression on different cell populations [11,43]. However, resting porcine T lymphocytes can express MHCII before activation [11,43], whereas human T cells only express MHCII when activated [44].

All the cytokines in the human Th1/Th2/Th17/Treg paradigm have porcine orthologs [36], however, it is suggested that IL-4 might play a different role in pigs [45]. The expression and frequency of immunoglobulins are quite similar (Table 2) except that IgD has not been demonstrated in pigs. Similar to humans, pigs have at least five IgG subclasses: IgG1, IgG2a, IgG2b, IgG3 and IgG4 [11]. Humans have two IgA heavy constant region genes (Cα) and therefore two subtypes of IgA designated IgA1 and IgA2 [46], whereas pigs only have one Cα gene and therefore only one class of IgA [46-48]. Circulating IgA is mostly bone marrow derived and monomeric in humans [49], while circulatory IgA in pigs is half dimeric IgA and half monomeric IgA [50]. The dimeric proportion of circulating IgA in the pig is, however, primarily derived from the intestinal synthesis and lymph. Due to the hepatic pIgR-mediated transcytosis of polymeric IgA (pIgA) to the bile, the dimeric IgA is thought to be relatively short-lived in the circulation [50]. The hepatic polymeric immunoglobulin receptor (pIgR)-mediated transcytosis of pIgA happens in both humans and pigs [50].

In women, IgA2 is known to be the predominant isotype subclass in the genital secretions [51] while this distinction cannot be made in the porcine FGT secretions.

When modeling genital infections and evaluating vaccine responses, the toll-like receptors (TLR) play a crucial role in recognition of the pathogens and induction of and controlling/directing the immune response. It has been shown that the porcine TLR system is very similar to that of humans [41]. In terms of cytokines such as the neutrophil chemokine IL-8, the coding gene carried by humans and pigs is an ortholog [41]. Furthermore, human- and porcine macrophages produce indoleamine 2,3-dioxygenase (IDO) in response to lipopolysaccharide (LPS) and Interferon gamma (IFN-ɣ) stimulation [36,41].

### 6.1. The genital mucosal immune response

The genital mucosal immune responses are of specific importance when using the pig as a model of human genital *C. trachomatis* infections. The genital immune response is challenged in the sense that it has to tolerate sperm, the semi-allogeneic conceptus and the commensal vaginal flora, while it must mount defense responses against sexually transmitted pathogens in order to eliminate them [52].

The genital immune system consists of both innate and adaptive factors. The innate system is primarily built by the epithelial barrier, the production of antimicrobial agents and cytokines by the epithelial cells and the innate immune cells [40,53]. Both innate and adaptive humoral mediators and immune cells in the genital immune system are regulated by progesterone and estradiol and therefore fluctuate through the menstrual or estrous cycles [53].

The epithelial cells in the FGT with interconnecting tight junctions play an important role in the immune protection by providing a strong physical barrier, transporting antibodies to the mucosal surface, secreting antibacterial compounds and by recruiting immune cells [54,55]. The sex hormones regulate the structural changes in the epithelium during the cycle. Under the influence of estrogen, the integrity and strength of tight junctions in the epithelial barrier, is significantly weakened in women [54,56]. The secretion of antimicrobial compounds is also suppressed during the midcycle in women [53,57].

To preserve an intact protective barrier, the genital mucosal immune response is often non-inflammatory to avoid inflammation-mediated injuries usually caused by phagocytic activity and complement activation [55]. Most of the antigens in the FGT are therefore met with mucosal tolerance [55].

#### 6.1.1. Distribution of immune cells in the genital tract tissue

The genital mucosa does not have immune inductive sites such as the nasal-associated lymphoid tissue or intestinal Peyer’s patches [55]. Thus, the genital mucosa lacks an organized center to disseminate antigen-stimulated B and T lymphocytes to the distinct sites of the mucosa. However, lymphoid aggregates (LA) are present in the female genital mucosa of both pigs [35] and humans [55] and leukocytes are dispersed throughout the mucosa of the FGT [58] as illustrated in Figure 2.

The LA are located in the basal layer of the endometrium close to the base of the uterine epithelial glands and built by a core of B cells surrounded by T cells and an outer layer of macrophages [58]. The T cells in the LA are primarily CD8^+^ T cells, however, CD4^+^ T cells are also present in variable numbers in the LA [58]. Both CD4^+^ and CD8^+^ T cells are found as intraepithelial lymphocytes and dispersed throughout the subepithelial tissue [58]. Aggregates of NK cells can also be found in the endometrium but they are placed in close contact with the luminal epithelium [58].

The leukocytes present in the FGT covers macrophages, dendritic cells, NK cells, neutrophils, B cells and T cells [53,59,60] with lymphocytes being the predominant immune cell type in both pigs and women [35,61,62]. The number of immune cells and the size of LA are under strong hormonal influence and fluctuate through the cycle [55,58] as summarized in Table 3.

**Table 3:** **Fluctuations in immune cells and antibody levels in the female genital tract during the hormonal cycles.** Both women and pigs show regional differences in the hormonal regulation of the genital immune system. The antibody fluctuations seem similar in women and pigs but the influx of neutrophils during estrus is specific for pigs. It should be noted that the porcine studies are rather old and only including few animals. LGT – Lower genital tract, UGT – upper genital tract

		**Women**	**Pigs**
LGT	Immune cells	Compared to the other regions of the female genital tract (FGT) the vaginal mucosa houses only few lymphocytes and antigen presenting cells (APC) [95]. The cervix, on the other hand, is an immunologic hotspot with the highest concentration of lymphocytes (both T cells and B cells) and APC [55,95].	The number of plasma cells in the vaginal mucosa has been shown to increase during estrous [32,67].
		No significant changes, but a slight increase in the number of immune cells in the secretory phase has been shown [57].	No significant changes was seen in the cervical mucosa [35], but a tendency was found, that the number of intraepithelial macrophages increased in estrous and that the number of lymphocytes, plasmacells and macrophages in the subepithelial tissue increased during estrous [32,35].
		The activity of cytotoxic CD8 T cell in the lower genital tract (LGT) is persistent during the cycle [96].	The cervix does not show infiltration by neutrophils during estrus [29,67,97].
	Antibody response	The total IgG and IgA levels on the mucosa are high after menstruation in the proliferative phase, decrease significantly around ovulation and keeps a medium level in the luteal phase [65,66,98–100].	The amount of antibodies on the mucosa has been shown to decrease during estrus/ around ovulation [67].
UGT	Immune cells	Only few neutrophils are present during the proliferative phase but the number increase towards the menses and are high during the menses [96]. Generally polymorphnuclear leukocytes, macrophages, NK cells and T cells accumulate in the endometrium in the luteal phase during high progesterone level [58,59,96] and the number of macrophages reaches maximum during menses [101].	The uterine mucosa shows an infiltration of neutrophils in proestrous and estrous [29,35,62,97] positively correlated to the estradiol levels [29].
		The lymphoid follicles, in the subepithelial tissue develop during the proliferative phase, reach the largest size during midcycle, remain large during the secretory phase and almost disappear at the menses [58,102].	Intraepithelial and subepithelial macrophages and lymphocytes are also more numerous during estrus and early diestrus [29,32] with the peak in number of lymphocytes during early diestrus [104].
		Activity of cytotoxic T cells in the mucosa is suppressed in the secretory phase [96].	There were no reportings on difference in size of the lymphoid aggregates during cycle [61].
		The number of APC in the fallopian tubes is significantly higher after ovulation in the luteal phase compared to the preovulatory follicular phase [103].	Studies have found either no variation in number of immune cells in the fallopian tube mucosa during the estrous cycle [97] or an increase in number of plasma cells and lymphocytes during estrous [32].
	Antibody response	The uterine secretions display the highest levels of IgG around the ovulation/midcycle [57].	Further studies are needed on the fluctuation of antibody levels in the upper porcine FGT.
		The Fallopian tubes show a response similar to the lower FGT with a lower level of antibodies around midcycle [57].	

#### 6.1.2. The humoral genital immune response

The immunoglobulins found in the FGT either have been locally produced by subepithelial plasma cells, or derived from the circulation [63]. Although IgG producing plasma cells can be found in the FGT [64], genital IgG is mainly derived from the circulation [63,65-67] and transported to the mucosal surface by mechanisms such as passive leakage, paracellular diffusion or receptor-mediated transport [63,65]. In contrast, genital IgM and IgA are primarily derived from the subepithelial plasma cells [65,68-70] with up to 95% of the porcine IgA being locally produced [71] and up to 70% of the IgA being locally produced in women [55]. When produced locally, the polymeric secretory IgA (sIgA) is actively transported across the mucosal epithelia cells by the polymeric immunoglobulin receptor (pIgR) [65,66]. The secretion of sIgA primarily takes place in the cervix due to the focused pIgR localization in the cervix in women [72]. The pIgR is also expressed in the uterus, but to a lesser extent and in variable levels due to hormonal regulation [55].

Usually, sIgA is the predominant isotype found in mucosal secretions, such as the intestinal fluid. However, in the secretions from the FGT, there is a greater proportion of IgG compared to sIgA [65,73-75].

The FGT humoral immune response is under strong hormonal influence during the menstrual or estrous cycle [57,74]. The cyclic fluctuations in the antibody levels are compared in Table 3. The information on cycle-dependent variations in the level of antibodies in pigs is sparse and more knowledge is needed within this area.

#### 6.1.3. Immunological differences of relevance for a *Chlamydia* model

The most important immunological difference with potential influence on *Chlamydia* models is the slightly different influx of immune cells in the porcine FGT, characterized by an increase in neutrophils during estrus. It should be kept in mind that this increased innate response during estrus could influence the establishment of infection.

## 7. The vaginal flora and pH

In women, the vaginal microflora is known to play an important role in the innate genital immune system by inhibiting the colonization of pathogens [76,77]. Lactobacilli and other lactic acid producing bacteria are particularly associated with equilibrium in the vaginal flora and inhibition of the growth of pathogens [76,78,79].

16S rRNA gene sequencing has allowed a thorough identification of the vaginal flora in women and the most common bacteria are: *Lactobacillus* spp*.*, *Staphylococcus* spp*.*, *Ureaplasma urealyticum*, *Corynebacterium vaginale*, *Streptococcus* spp*.*, *Peptostreptococcus* spp*.*, *Gardnerella vaginalis*, *Bacteroides* spp*.*, *Mycoplasma* spp*.*, *Enterococcus* spp*.*, *Escherichia coli*, *Veillonella* spp*.*, *Bifidobacterium* spp*.* and *Candida* spp*..* However, the species composition can be very different between individuals and during the menstrual cycle [52,76,79]. In women, the lactic acid producing bacteria play an important role by contributing to an acidic environment with a pH of 3.5–5 [52].

In healthy pigs the vaginal flora has been characterized by culture dependent methods and was found to include both aerobic and anaerobic bacteria with the most prominent being the following: *Streptococcus* spp., *E. coli*, *Staphylococcus* spp., *Corynebacterium* spp., *Micrococcus* spp. and *Actinobacillus* spp. [80]. Based on our genetic screening of vaginal swabs from Göttingen Minipigs, it is evident that the above mentioned bacteria are present, but not dominating. *Streptococcus* spp. constituted on average 1.4% on the vaginal flora, *E. coli* 3.7%, and *Staphylococcus* 0.4%. Furthermore, we found that the vaginal flora was not dominated by lactobacillus as in humans. *Lactobacillaceae* constituted on average 3.9% of the total vaginal flora in Göttingen Minipigs. The vaginal flora in Göttingen Minipigs seemed to be dominated by the following: unclassified genera belonging to Gammaproteobacteria, unclassified genera from Clostridiales, *Yersinia*, *Paenibacillus*, *Listeria*, *Syntrophus*, *Heliobacterium*, *Faecalibacterium*, *Kineococcus* and *Proteus* (unpublished data).

An old study showed that the FGT mean pH in estrus in pigs is 7.02 in the oviduct, 6.98 in the uterus, 7.49 in the cervix and 6.61 in the vagina [81]. Our own data, based on vaginal pH measurements with a pH electrode (Mettler-Toledo InLab® Surface Electrode, Sigma-Aldrich Broendby, Denmark), confirmed that the vaginal pH is just around neutral (~7) in both prepubertal and sexually mature Göttingen Minipigs.

## 8. Important differences between rodents and minipigs

The primary aim of this review was to compare the female reproductive physiology of humans and pigs, however, as a concluding section, we found it important to highlight where the minipig shows significant differences to the commonly used murine model in *Chlamydia* research. Similar comparisons of humans and mice has been done elsewhere [4,82,83], and only main points will be included here.

The reproductive cycle is significantly shorter in mice, having a 4–5 day cycle due to the lack of progesterone-producing *corpora lutea* and thereby a luteal phase, if no coital stimulation occurs [84]. Anatomically, the murine uterus is bicornuate and much smaller than the porcine and human ones [83]. Histologically, the vagina displays keratinized squamous epithelium during estrus, whereas porcine and human epithelium does not keratinize [83].

Within the immune system, the composition of circulating leukocytes is significantly different with a lower percentage of neutrophils and a corresponding higher abundance of lymphocytes in mice compared to pigs and humans [82]. Furthermore, and importantly for the *Chlamydia* model, murine macrophages do not produce IDO in response to LPS and IFN-ɣ stimulation, by contrast humans and porcines do [36,41]. Furthermore, murine macrophages produce nitric oxide (NO) in response to stimulation with LPS, whereas human and porcine macrophages do not [36]. There is also a great difference in the expression of cytokines such as IL-8, a strong neutrophil chemokine expressed in pigs and humans, but not in mice. In mice keratinocyte-derived chemokine and macrophage inflammatory protein-2 are considered to be the IL-8 counterpart [41].

In the FGT, the influx of immune cells happens slightly differently in mice, compared to pigs and humans. In the murine endometrium an influx of leukocytes is seen in the proestrus, during estrus the leukocytes are almost absent, during metestrus they are prominent and during diestrus an infiltration is seen [83]. The fluctuations in antibody levels in the murine FGT shows a similar pattern for IgG, with a lower level during estrus, while for IgA, it is opposite that of pigs and women, with mice having a higher level during estrus [85].

## 9. Conclusions

This comparison of the porcine and human FGT reveals clear similarities and gives an understanding of the differences between the species. Despite the bicornuate porcine uterus with a urogenital sinus and cervical pulvini, the anatomical and morphological construction and proportion of layers with cyclic alterations is very similar in humans and pigs. The hormonal cycles are closely related, only differing slightly in cycle duration, and origin of luteolysing hormone. The general immune system and the immune system associated with the FGT show great similarities. The antibody levels on the genital mucosa shows similar cyclic fluctuations in pigs and women, but the immune cell infiltration in the genital mucosa differs slightly between women and pigs, namely in the influx of neutrophils in the porcine endometrium during estrus. The porcine vaginal flora differs from the human by not being dominated by lactobacilli and the vaginal pH is slightly higher in pigs than in women.

It is difficult to tell the exact significance of the differences and similarities between the FGT in women and pigs and interpretation of data from animal models should always be done with caution. The similarities found in this review, however, suggest that the pig adds a greater predictive value to FGT studies than what can be achieved by studies in rodent models. Non-human primates is the species most closely related to humans, but ethical concerns and the relative ease of working with pigs propose the pig to be an advantageous model of human reproductive disorders such as *C. trachomatis* infection.

## 10. Abbreviations

APC: Antigen presenting cell
FGT: Female genital tract
IDO: Indoleamine 2,3-dioxygenase
IFN-ɣ: Interferon gamma
Ig: Immunoglobulin
LA: Lymphoid aggregates
LGT: Lower genital tract
LPS: Lipopolysaccharide
MHC: Major Histocompatibility complex
NHP: Non-human primates
NO: Nitric Oxide
PGF-2α: Prostaglandin-F2α
pIgR: Polymeric immunoglobulin receptor
pIgA: Polymeric immunoglobulin A
sIgA: Secretory Immunoglobulin A
SLA: Swine leukocyte antigen
TLR: Toll-like receptor
UGT: Upper genital tract
